# Effects of rainfall intensity on runoff and nutrient loss of gently sloping farmland in a karst area of SW China

**DOI:** 10.1371/journal.pone.0246505

**Published:** 2021-03-18

**Authors:** Yiwen Yao, Quanhou Dai, Ruxue Gao, Yixian Gan, Xingsong Yi

**Affiliations:** Institute for Forest Resources & Environment of Guizhou, College of Forestry, Guizhou University, Guizhou University Institute of Soil Erosion and Ecological Restoration, Guiyang, China; Soil and Water Resources Institute ELGO-DIMITRA, GREECE

## Abstract

Nutrient losses from sloping farmland in karst areas lead to the decline in land productivity and nonpoint source pollution. A specially tailored steel channel with an adjustable slope and underground hole fissures was used to simulate the microenvironment of the "dual structure" of the surface and underground of sloping farmland in a karst area. The artificial rainfall simulation method was used to explore the surface and underground runoff characteristics and nutrient losses from sloping farmland under different rainfall intensities. The effect of rainfall intensity on the nutrient loss of farmland on karst sloping land was clarified. The results showed that the surface was the main route of runoff and nutrient loss during the rainy season on sloping farmland in karst areas. The influence of rainfall intensity on the nutrients in surface runoff was more substantial than that on underground runoff nutrients. Nutrient loss was more likely to occur underground than on the surface. The losses of total nitrogen, total phosphorus, and total potassium in surface and underground runoff initially increased and then gradually stabilized with the extension of rainfall duration and increased with increasing rainfall intensity and the amount of nutrient runoff. The output of nutrients through surface runoff accounted for a high proportion of the total, and underground runoff was responsible for a low proportion. Although the amount of nutrients output by underground runoff was small, it could directly cause groundwater pollution. The research results provide a theoretical reference for controlling land source pollution from sloping farming in karst areas.

## Introduction

**Fig 1:**
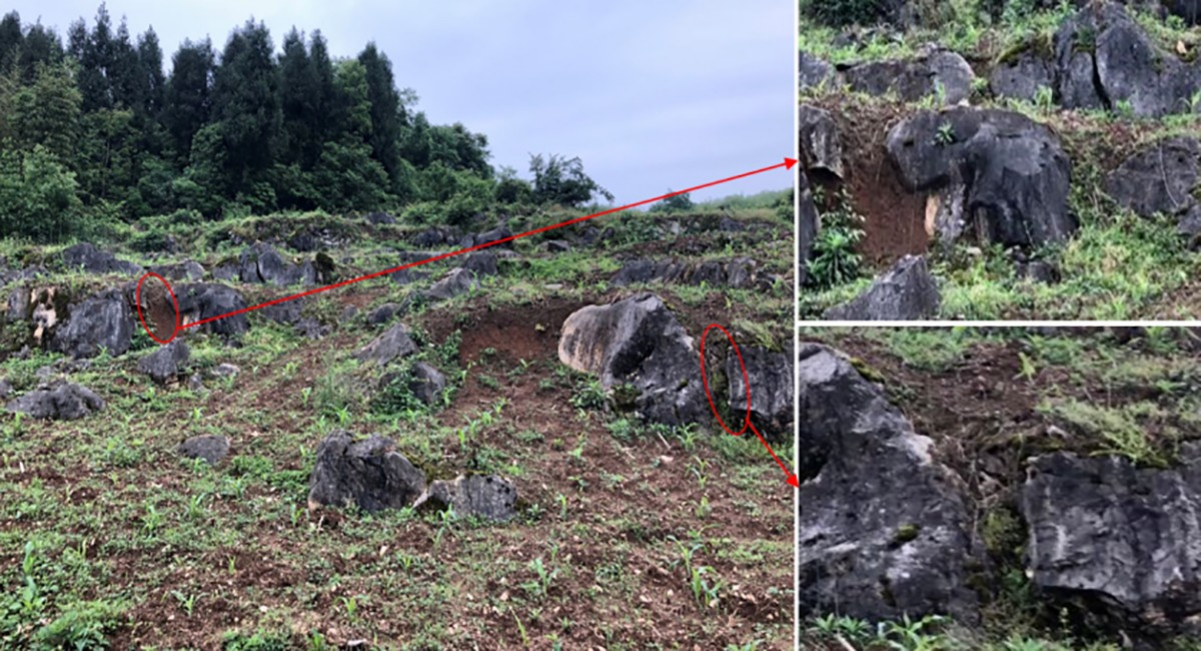
Underground fissure of slope farmland in karst area.

Soil nutrient loss is a universal phenomenon worldwide [1], and how to control and prevent nutrient loss is also a focal point of international research [2, 3]. As one of the largest concentrated karst areas globally [4], the karst area in Southwest China [5] has a low surface soil thickness and high rock exposure rates due to rocky desertification with severe soil erosion on sloping farmland. Sloping farmland is one of the critical agricultural land-use types in China [6], of which gently sloping farmland (15~25°) accounts for 23.8% of the country’s total farmland area. In recent years, the demand for food has increased as the population has grown. There are contradictions in improving land productivity and protecting arable land. The use of many chemical fertilizers and pesticides and unreasonable farming methods have caused substantial soil erosion [7], which directly leads to the loss of soil nutrients in sloping farmland. Nutrient loss causes a decline in the productivity of sloping farmland [8]; on the other hand, it also causes eutrophication of surface water bodies [9, 10]. The karst area in southern China covers 540,000 square kilometers, accounting for 55% of China’s total karst area. This area is an integral part of the terrestrial ecosystem in China. As the center of the southwest karst region, Guizhou Province covers 150,000 square kilometers with bare carbonate rocks, accounting for 73.6% of its total land area. This type of soluble rock will dissolve under long-term natural processes, impacting the surface and underground hydrogeological structures [11]. Consequently, soil nutrients of sloping farmland are lost from the ground through surface runoff and karst cracks, pipes, and holes (Fig 1) [12, 13]. In addition, a large area of exposed bedrock in karst areas limits the size of arable land and causes more severe soil erosion than in non-karst regions. The path of soil nutrient loss is even more complicated.

Soil nutrients in sloping farmland can be lost through volatilization [14], vertical leaching [15], runoff, and sediment transport [13], and runoff is the main mechanism. There are two ways for soil nutrients to enter runoff: nutrients are dissolved in the soil solution and enter the surface runoff through water exchange, and nutrients are adsorbed on the surface of soil particles and enter the surface runoff through desorption or are accompanied by eroded sediment. The main factors affecting soil nutrient loss are rainfall intensity [16], slope [7], slope length [17], flow [18], farming methods [19, 20] and vegetation cover [21]. The methods used by researchers to investigate nutrient loss mainly focus on location monitoring [22] and artificial rainfall simulation [23]. Some scholars have used short pulses [24] and element tracing [25] methods to study the influence of rainfall in karst areas on soil nutrient migration paths with runoff. The soil nutrient loss in karst areas is different from that in other areas, and nutrients are affected by surface and underground runoff [26]. Gao et al. [13] found that the nutrients of sloping farmland in karst areas are mainly lost through groundwater flow when the rainfall intensity is low. The existence of underground pores and cracks allows nutrients and water to migrate into the groundwater system through soil infiltration and the deep part of the rock-soil boundary. Nevertheless, the impact on nutrient loss is not obvious [23].

Most of the research on nutrient loss in karst sloping farmland focuses on evaluating the surface loss law. Limited by the complicated geological environment of the karst area, field tests are strict. The existing research methods are limited, and there are still some problems, such as how to determine underground runoff and its nutrient loss changes and the ratio of underground runoff nutrients to total runoff. The mechanism by which rainfall intensity affects surface and subsurface runoff and nutrient loss is still unclear. Therefore, this paper takes 15° sloping farmland in karst areas as the research object and uses variable slope steel troughs with perforated floors to simulate the "double-deck" spatial structure of the surface and underground sloping farmland in rocky desertification fields in karst areas to (1) reveal the influence of rainfall intensity on runoff from karst sloping farmland and nutrient losses from runoff and (2) explore the surface and underground runoff and its nutrient loss ratio under different rainfall intensities. This study provides a theoretical reference for controlling ground source pollution in light rocky desertification sloping farmland in karst areas.

## Materials and methods

### Test materials

**Fig 2:**
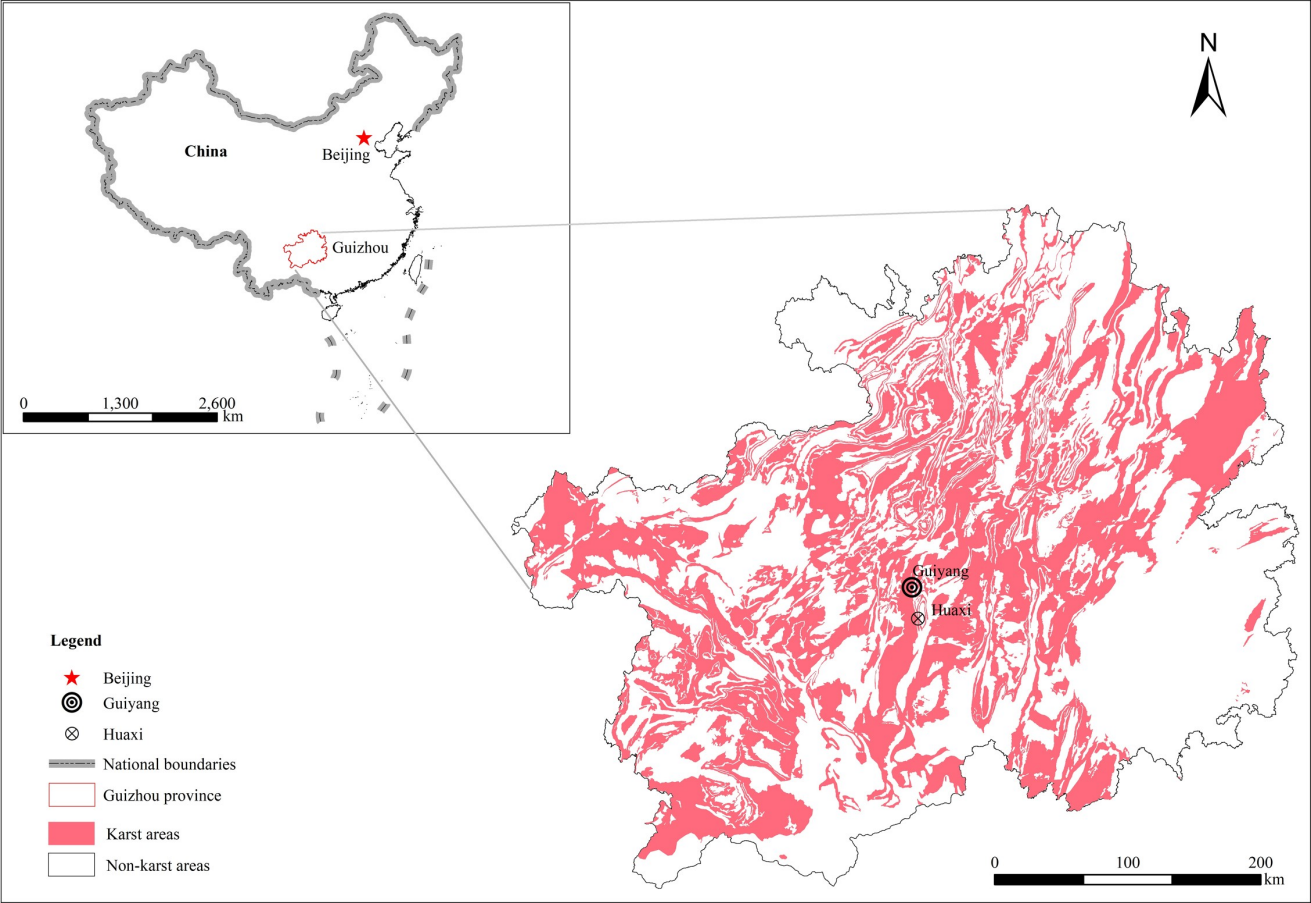
Location of the study area (Huaxi District, Guiyang city, Guizhou Province, China).

The experimental soil was derived from sloping farmland soil in a typical karst rocky desertification area located in Qingyan town, Huaxi District, Guiyang city, Guizhou Province (26° 19’ 17" N, 106° 39’ 18" E) (Fig 2) (http://www.resdc.cn/DataList.aspx). Guiyang is located in a subtropical humid monsoon climate. The annual average number of sunshine hours is approximately 1062.0 h. The average yearly temperature is approximately 15.1°C, the annual average relative humidity is 77%, the average annual precipitation is 1100~1300 mm, and the frost-free period is approximately 270 days. Calcareous soil was collected from the 0~20 cm soil layers of cultivated land. Calcareous soil refers to the soil developed from carbonatite weathering materials, and carbonate rocks are widespread in Southwest China. Limestone soil is widely disseminated in the karst areas of Guizhou Province. The limestone soil area in this province is 6.8×103 km^2^, accounting for 7.9% of the cultivated land in this area [27]. The soil particle composition and original soil nutrients are shown in Table 1. The test soil was not sieved, and impurities such as roots and stones were removed from the soil. Large soil lumps were dispersed and evenly mixed for use after natural air drying.

**Table 1:** Basic properties of the test soil.

Soil type	Particles mechanical composition (%)			TN(g/kg)	TP(g/kg)	TK(g/kg)
	Sand	Silt	Clay (<0.002mm)			
	(0.02-2mm)	(0.002–0.02mm)				
Calcareous soil	37.34±3.33	47.53±2.08	15.03±1.02	1.72±0.19	1.69±0.14	8.47±1.05

### Experimental rainfall setup

**Fig 3:**
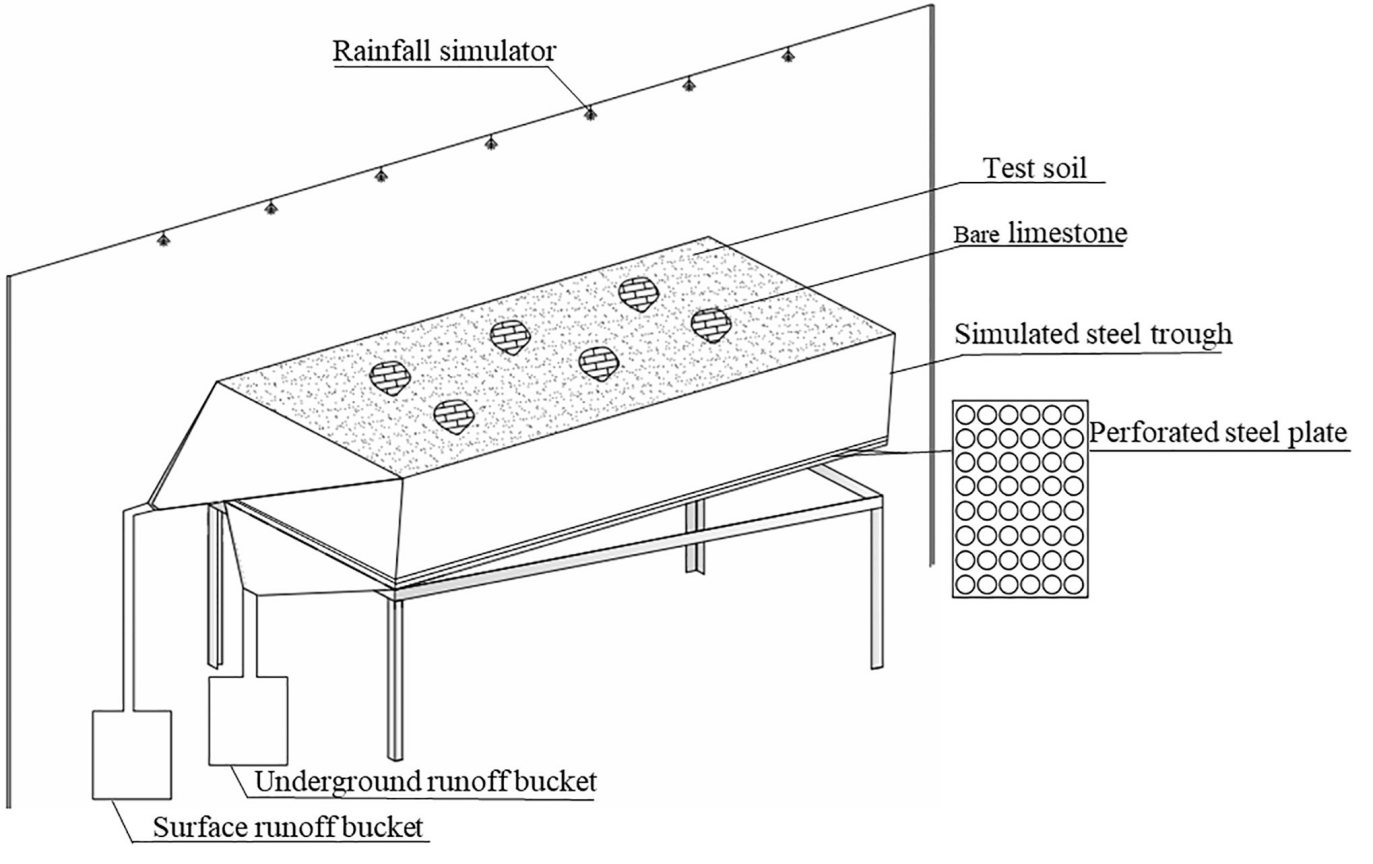
Sketch of the experimental apparatus.

The test instrument consisted of a portable automatic artificial rainfall simulator and a self-designed variable slope steel trough (Fig 3). The artificial rainfall simulator was the QYJY-501 model, the rainfall height was 6 m, and the effective rainfall area was 6.5 m × 6.5 m. The uniformity was >85%, the end speed of raindrops met the characteristics of natural rainfall, the adjustment accuracy was 7 mm·h^-1^, and the adjustment change time was less than 30 s. The rainfall intensity could be controlled automatically or manually by the controller, and the rainfall duration could be adjusted arbitrarily.

The length, width, and depth of the variable slope steel channel were 4 m, 1.5 m, and 0.35 m, respectively. The slope of the steel channel was adjusted to 0~45°. There were two steel plates at the bottom of the steel channel, and each steel plate had 192 evenly distributed holes with diameters of 5 cm. The underground hole (crack) size was adjusted by changing the overlapping area of the two steel plate holes, and the adjustment range was 0~8%. Enormous damage was created when the holes completely overlapped, and the smallest crack was created when the holes were completely staggered. The steel trough was provided with collecting channels for surface and underground flows.

### Experimental design

This study took sloping farmland with mild rocky desertification as the research object. By simulating the surface and underground dual structure of the sloping farmland in this area, the surface and underground runoff loss status and nutrient loss characteristics could be studied. The exposed rate of bedrock in the experiment (20%), slope (15°), and crack degree (1%) were fixed factors, and the rainfall intensities (30, 50, 70, 90 mm·h^-1^) were taken as experimental factors. The bedrock exposure rate referred to the ratio of the bare bedrock area to the slope horizontal projection area [28], which could be divided into three levels: no rocky desertification (10%), potential rocky desertification (20% and 30%), and mild rocky desertification (40% and 50%). The research team conducted a field survey of 160 sample plots in the main karst distribution areas of Guizhou Province and combined it with literature analysis. The exposed rate of bedrock on sloping farmland in the Guizhou karst area was 10%~30%. The slope was within the range of 10°~25°; the most extensive fissure degree was 5.98% [29, 30]. The method of adjusting the clearance was first to calculate the maximum chord length of the overlapping area of the bottom hole when the release of the crack was at the designed level, and the chord length of the overlapping hole area was adjusted to the design level through the rocker arm [31]. The rainfall intensity was based on the erosive rainfall index of sloping karst farmland proposed by Zhang et al. [32]. Combined with the previous research results of the research group [23, 28], it was found that the erosive rainfall of sloping farmland in the karst area occurred only when the surface runoff and sediment yield were above 30 mm·h^-1^. The rainfall intensity was divided into three types: light rainfall intensity (30 mm·h^-1^), moderate rainfall intensity (50~70 mm·h^-1^), and heavy rainfall intensity (90 mm·h^-1^).

Carbonate rock with a diameter of ≥35 cm was collected and randomly arranged in a steel channel filled with soil, and the rate of bedrock exposure was then designed. The soil filling of the steel trough consisted of three 10-cm-thick layers, and according to the measured soil compactness in the field, the soil compaction was 1070, 760, and 410 kPa from bottom to top. When the soil layer’s total depth reached 30 cm, unique wooden boards were used to compact the contact between the soil and the edge of the rock and the steel channel to reduce edge effects. Before the experiment, the slope of the steel tank was adjusted to the test level. Light rain (<15 mm·h^-1^) was initially used. Rain was stopped when the ground or underground exhibited runoff, and the experiment was started after standing for one hour. The duration of each rainfall event was 30 minutes, which was repeated three times at each rainfall intensity. After each rain event was over, the soil in the steel trough was replaced with fresh soil. Once the design requirements were met, the next rainfall event began. After the experiment started, a 500 ml polyethylene bottle was used to collect water samples of surface runoff and underground pore (fracture) flow every 3 minutes to determine the contents of total nitrogen, total phosphorus, and total potassium in the runoff water sample. The remainder of the runoff was collected in the runoff vat and measured as runoff. The collected water sample was adjusted with sulfuric acid so that its pH value did not exceed 2, and it was stored in a refrigerator not exceeding 4°C for testing.

### Analytical technics

The total nitrogen (TN) in water was determined by potassium persulfate oxidation ultraviolet spectrophotometry, total phosphorus (TP) was determined by potassium persulfate oxidation molybdenum antimony resistance colorimetry, and total potassium (TK) was determined by atomic absorption spectrometry. The detailed steps followed the "Water and Wastewater Monitoring and Analysis Method" [33]. The water nutrient content was determined by measuring the water nutrient content of a blank sample and subtracting the result from the water nutrient content measured from a water flow sample.

### Data handling

1. Runoff modulus (*M*)
  $$M=\frac{Q}{A}$$
  In the formula, M represents the runoff modulus (m^3^/s·km^2^) (the amount of runoff per unit time per unit basin area); Q represents the average flow (m^3^/s); and A represents the drainage area (km^2^).
2. Nutrient loss ratio (*R_X_*)
  $$R_{X}=\frac{N_{t}}{W}$$
  In the formula, *R_X_* represents the percentage of nutrient output through surface or groundwater flow (%), *N_t_* represents the amount of surface or underground nutrient loss (mg), and *W* represents the total nutrient loss (mg).
3. Loss modulus of soil nutrients (*K*)
  $$K=\frac{N_{t}}{T\times S}$$
  In the formula, K represents the runoff nutrient loss modulus (mg/h/m^2^) (that is, the amount of nutrient loss through water flow per unit horizontal projection area per unit time, which is an index of nutrient loss intensity); *N_t_* represents the amount of surface or underground nutrient loss (mg); and S represents the horizontal projection area of the 15° steel channel floor (m^2^).

Excel 2019 software was used for statistical data analysis. SPSS 20.0 (Statistical Package for the Social Science) software was used to analyze the difference and correlation between different treatments, and Origin 2018 was used for mapping.

## Results

### Runoff output process and distribution characteristics under different rainfall intensities

**Fig 4:**
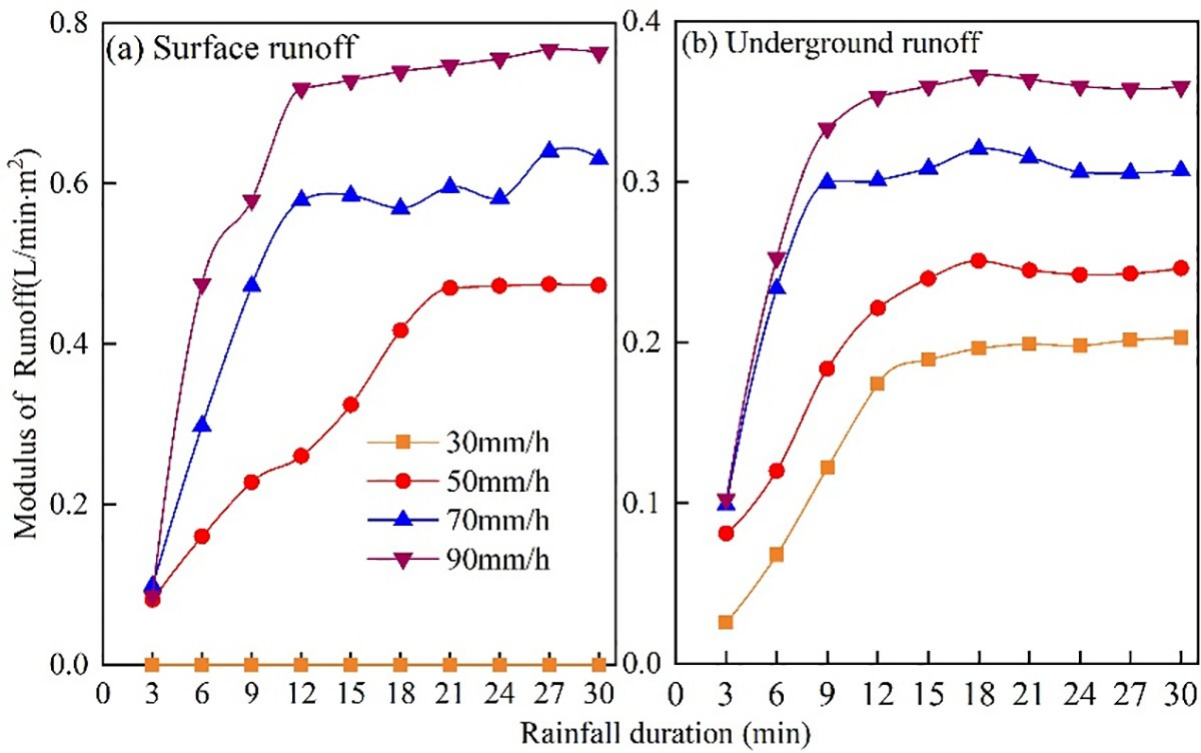
Variation of runoff with rainfall duration under different rainfall conditions.

Due to the unique "dual structure" of the karst area, surface runoff will form down the slope after rainwater falls on sloping farmland, and underground runoff will also be lost through cracks. The changes to surface and subsurface runoff at different rainfall durations and rainfall intensities are plotted in Fig 4. There was no surface runoff under light rainfall intensity (30 mm·h^-1^), and the rainfall intensity was never less than 50 mm·h^-1^. Initially, surface runoff was present, and surface and underground runoff increased with increasing rainfall intensity. Under different rainfall intensities, the runoff showed a trend of first increasing and then gradually stabilizing with the rainfall duration.

**Fig 5:**
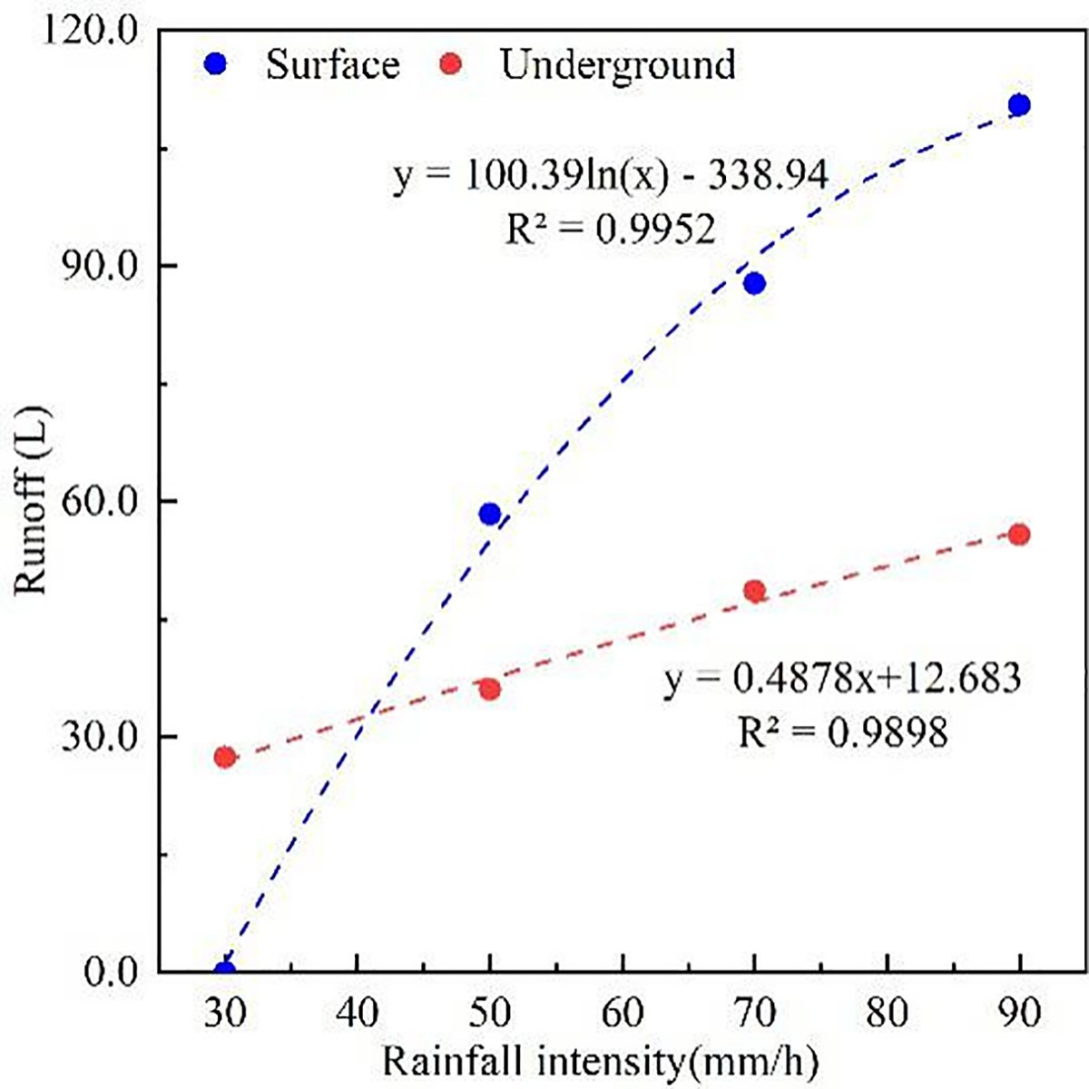
Linear regression equation of rainfall intensity and runoff.

The maximum surface and underground runoff occurred 12~15 minutes after rainfall. After 15 minutes of rain, the surface runoff modulus was maintained within the range of 0.09~0.77 L·min^-1^·m^2^ at 90 mm·h^-1^, and the underground runoff modulus under the same rainfall intensity was from 0.10~0.37 L·min^-1^·m^2^ after this period. The characteristics of runoff changes under different rainfall intensities are shown in Table 2. With the increase in rainfall intensity, surface runoff, underground runoff, and total runoff all showed significant increasing trends, with the highest runoff occurring under a rainfall intensity of 90 mm·h^-1^, and significant differences occurred between different rainfall intensities (*P*<0.05). During the 30 minutes of rainfall, surface runoff varied from 58.37±1.90 mm to 110.5±7.84 mm, and the underground runoff ranged from 27.41±0.88 mm to 55.74±1.68 mm. The surface runoff was significantly greater than the underground runoff. With the increase in rainfall intensity, the proportion of surface runoff in the total runoff gradually increased, with an average increase of 64.22%, indicating that the karst sloping farmland runoff was mainly through surface erosion. The proportion of underground runoff to the total runoff averaged 35.78%, meaning that underground decay should not be ignored. From the runoff modulus perspective, the surface runoff was higher than the underground runoff, and it increased with increasing rainfall intensity. Regression analysis of runoff and rainfall intensity (Fig 5) showed that rainfall intensity had a logarithmic relationship with surface runoff but a linear relationship with underground runoff, with R^2^ values of 0.9952 and 0.9898, respectively.

**Table 2:** Surface and underground runoff loss characteristics under different rainfall intensities.

Rainfall intensity (mm/h)	Surface		underground		Total runoff (L)	Proportion of surface runoff (%)
	Runoff (L)	Runoff modulus	Runoff (L)	Runoff modulus		
		(m^3^/s·km^2^)		(m^3^/s·km^2^)		
30	0	0	27.41±0.62d	2.63d	27.41d	0
50	58.38±0.13c	5.6c	36.04±1.23c	3.46c	94.41c	61.83
70	87.76±1.67b	8.42b	48.60±3.04b	4.66b	136.36b	64.36
90	110.5±8.14a	10.6a	55.74±2.81a	5.35a	166.24a	66.47

In the same column, there was a significant difference among different small letter groups (*P*<0.05), but there was no significant difference between the same small letter group (*P*> 0.05); the same is true below.

### Nutrient loss of runoff under different rainfall intensities

**Fig 6:**
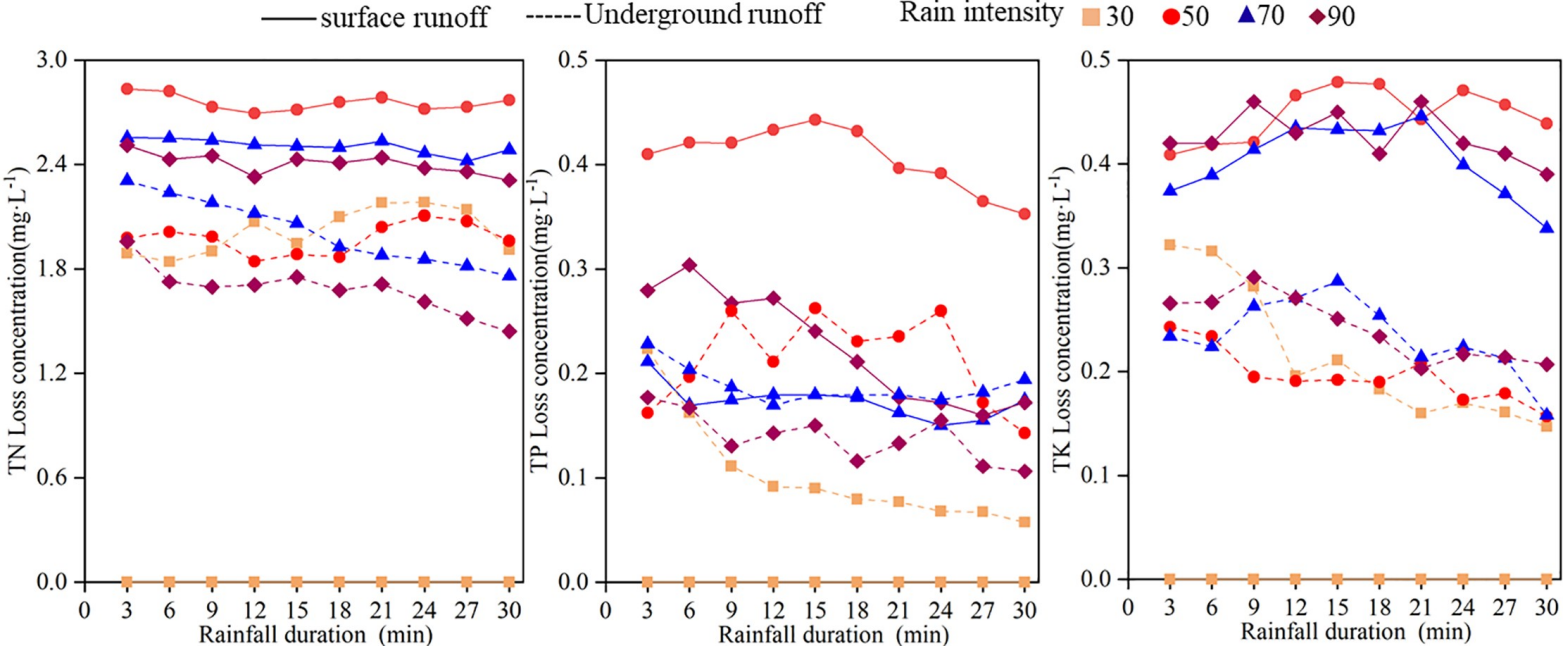
Variation of runoff nutrient concentrations with rainfall duration.

**Fig 7:**
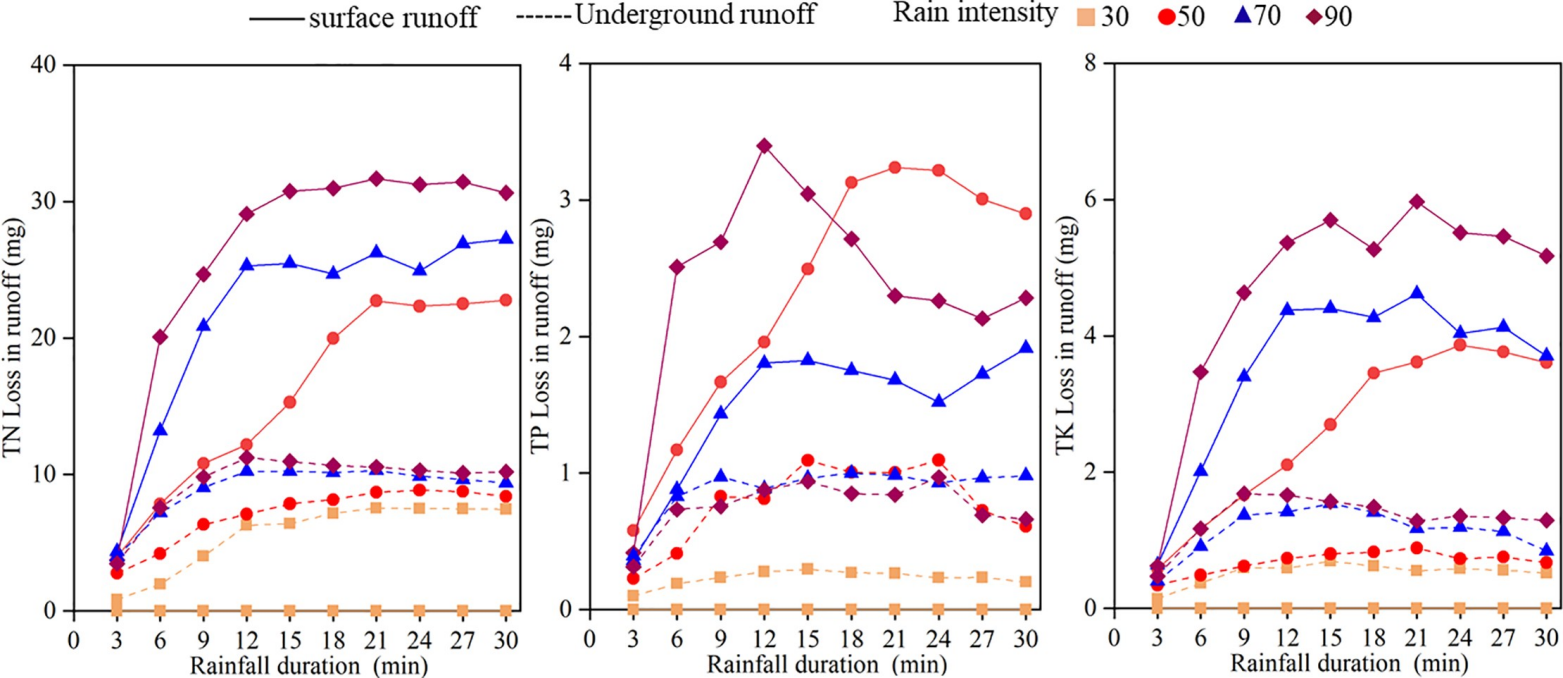
Variation of runoff nutrient and diversion loss with rainfall duration.

The nutrient concentrations and losses in surface and underground runoff with rainfall time under different rainfall intensities were plotted as graphs (Figs 6 and 7). The concentrations in surface runoff fluctuated with the duration of runoff. Under different rainfall intensities, the underground runoff concentrations presented a downward trend with the extension of rainfall time, but the initial erosion effects were not considered. The TP concentration significantly fluctuated throughout the rainfall process, indicating a slightly decreasing trend. With increasing rainfall time, the amount of nutrient loss over sloping farmland in karst areas first increased rapidly and then tended to gently increase (Fig 7), which was similar to the changes in runoff with rainfall duration. During the entire rainfall process, the time for each nutrient to reach its peak was slightly different under the different rainfall intensities. Under rainfall intensities of 70 and 90 mm·h^-1^, all nutrients reached their peak at 12 minutes of rainfall, and the time of nutrient diversion loss reached its peak at 18~21 minutes under a rainfall intensity of 50 mm·h^-1^.

The average concentrations of TN, TP, and TK in surface and underground runoff under different rainfall intensities are shown in Table 3. The average nutrient concentration of surface runoff was higher than that of underground runoff, and the average concentration significantly differed under different rainfall intensities (*P*<0.05). Among them, the average concentrations of TN and TK exhibited notable differences. Under heavy rain (90 mm·h^-1^), the average losses of TN and TK on the surface were 2.41 mg·L^-1^ and 0.43 mg·L^-1^, respectively, and the TN and TK losses underground were 1.68 mg·L^-1^ and 0.24 mg·L^-1^, respectively. The average TN concentration loss on the surface was 1.4 times that underground, and the TK concentration loss was 1.8 times that underground. The average TP concentration on the surface and underground did not change significantly (except for 50 mm·h^-1^ on the surface) and undulated. The reason for this result may be that TP is readily retained by the soil and has a weak migration ability in soil, resulting in TP in the runoff. The TP exhibited a small change in content.

**Table 3:** Surface and underground runoff loss characteristics under different rainfall intensities.

Type	Rainfall Intensity (mm/h)	Average loss concentration		Total loss		Nutrient loss modulus	
		(mg/L)		(mg)		(mg/h /m^2^)	
		surface	Underground	surface	Underground	surface	Underground
TN	30	0	2.02a	0	56.68c	0.00	19.58
	50	2.76a	1.98b	160.44c	71.15b	55.42	24.58
	70	2.51b	1.88a	219.18b	90.07a	75.71	31.11
	90	2.41c	1.72c	264.28a	94.93a	91.29	32.79
TP	30	0	0.10c	0	2.31c	0.00	0.80
	50	0.41a	0.21a	23.35a	7.82b	8.07	2.70
	70	0.17b	0.19a	14.90b	7.96a	5.15	1.75
	90	0.20b	0.16b	23.75a	8.53b	8.20	2.95
TK	30	0	0.21c	0	5.25d	0.00	1.81
	50	0.45a	0.20c	26.52c	6.86c	9.16	2.37
	70	0.40c	0.22b	35.59b	11.39b	12.29	3.93
	90	0.43b	0.25a	47.18a	13.31a	16.30	4.60

The nutrient loss and nutrient loss modulus in surface and underground runoff increased with increasing rainfall intensity as a whole (Table 3), and the difference in nutrient loss between different rainfall intensities was significant (*P*<0.05). Under a rainfall intensity of 50~90 mm·h^-1^, the loss of surface TN was 160.44~264.28 mg, and the failure of underground TN ranged from 71.15~94.93 mg. It was evident that the loss of TN from the surface was significantly higher than that of underground TN; TP and TK exhibited patterns similar to TN. From the nutrient loss modulus perspective, the surface nutrient modulus was higher than the underground nutrient modulus. The surface TP nutrient loss modulus under the same rainfall intensity (50 mm·h^-1^) was three times the underground TP loss modulus; on the other hand, the TN nutrient loss modulus was significantly higher than that of TK and TP. Under heavy rainfall intensities (90 mm·h^-1^), the surface nutrient modulus of TN was 91.29 mg·h^-1^·m^-2^, which was 11.1 times that of TP (8.20 mg·h^-1^·m^-2^) and 5.6 times that of TK (16.30 mg·h^-1^·m^-2^). Regression analysis of rainfall intensity and nutrient loss (Table 4) revealed that the rainfall intensity and surface and underground nutrient loss were logarithmic functions. Nevertheless, the R^2^ values of TN and TK were significantly higher than that of TP.

**Table 4:** Fitting regression equation of rainfall intensity and runoff nutrient loss.

position	index	Regression equation	R^2^
Surface	TN	y = 240.11ln(x) - 803.17	R^2^ = 0.9763
	TP	y = 36.661ln(x) - 69.001	R^2^ = 0.6296
	TK	y = 41.967ln(x) - 141.19	R^2^ = 0.9856
Underground	TN	y = 36.661ln(x) - 69.001	R^2^ = 0.9746
	TP	y = 5.2407ln(x) - 14.375	R^2^ = 0.7087
	TK	y = 0.1436x + 0.5857	R^2^ = 0.9637

### Nutrient loss ratio

**Fig 8:**
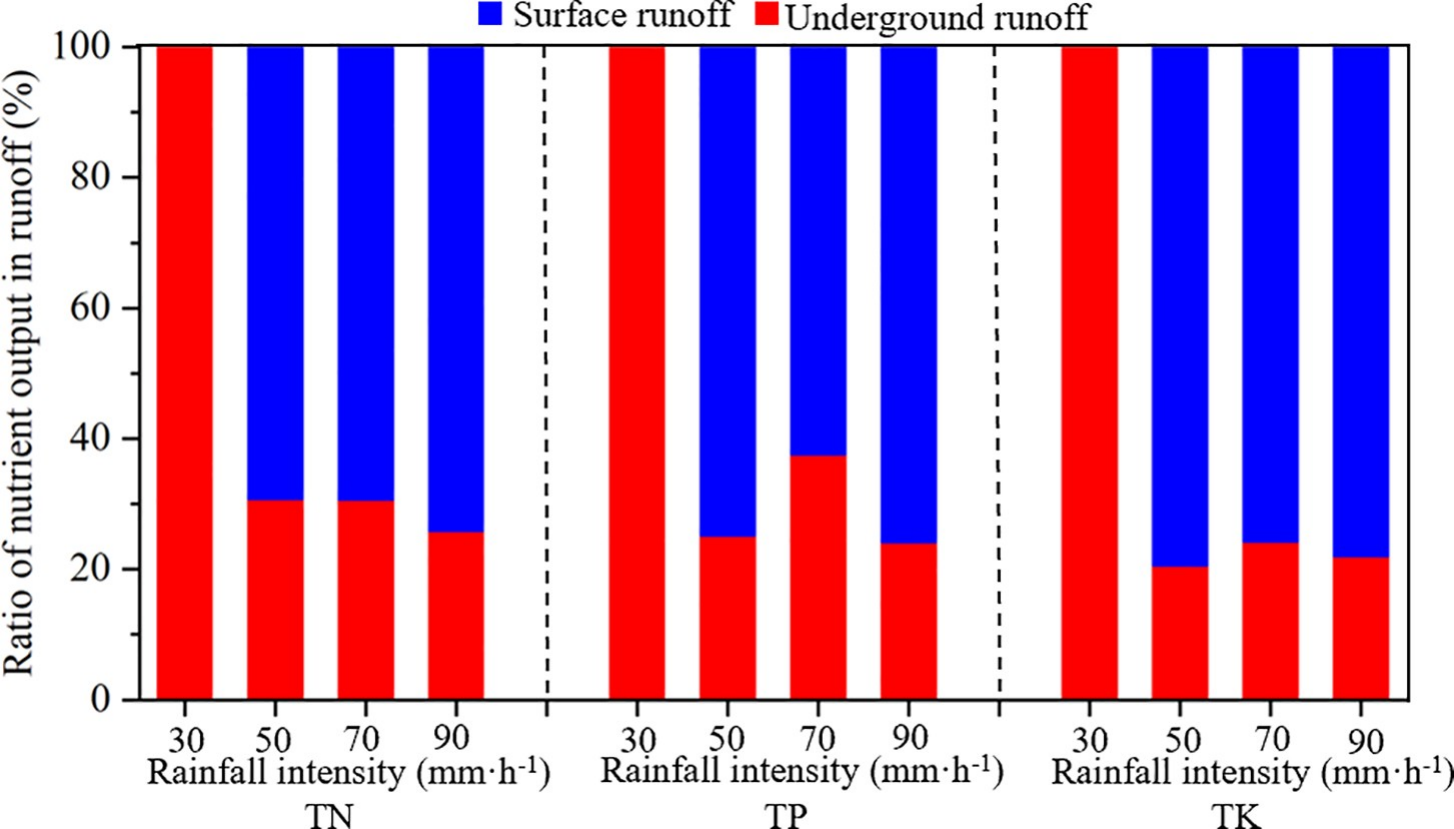
Proportion of nutrient loss in surface and underground runoff.

The movements of surface runoff and groundwater are affected by different external factors, resulting in significant differences in nutrient loss rates. Fig 8 shows that the nutrient loss rate through the underground fissure flow reached 100% with light rainfall intensity (30 mm·h^-1^). As the rainfall intensity increased, the surface runoff loss rate gradually increased. The loss rates of TN, TP, and TK in surface runoff under moderate rainfall intensity (50 mm·h^-1^) were 69.28%, 74.91%, and 79.45%, respectively, and the loss rates under heavy rainfall intensity (90 mm·h^-1^) were 74.11%, 75.93%, and 78.00%, respectively. The surface runoff loss rate increased with increasing rainfall intensity. The average loss of TN in surface runoff accounted for 70.94% of the total loss, that of TP was 71.10% and that of TK was 77.73%. The losses of TN, TP, and TK in underground runoff accounted for 29.06%, 28.90%, and 22.27% of the total loss, respectively. Although more nutrients were lost through surface pathways, the proportion of underground runoff loss reached 1/3 of the total loss, which shows that the loss of nutrients through underground tracks was also very severe.

### Effects of rainfall intensity and runoff on nutrient loss in sloping farmland

**Fig 9:**
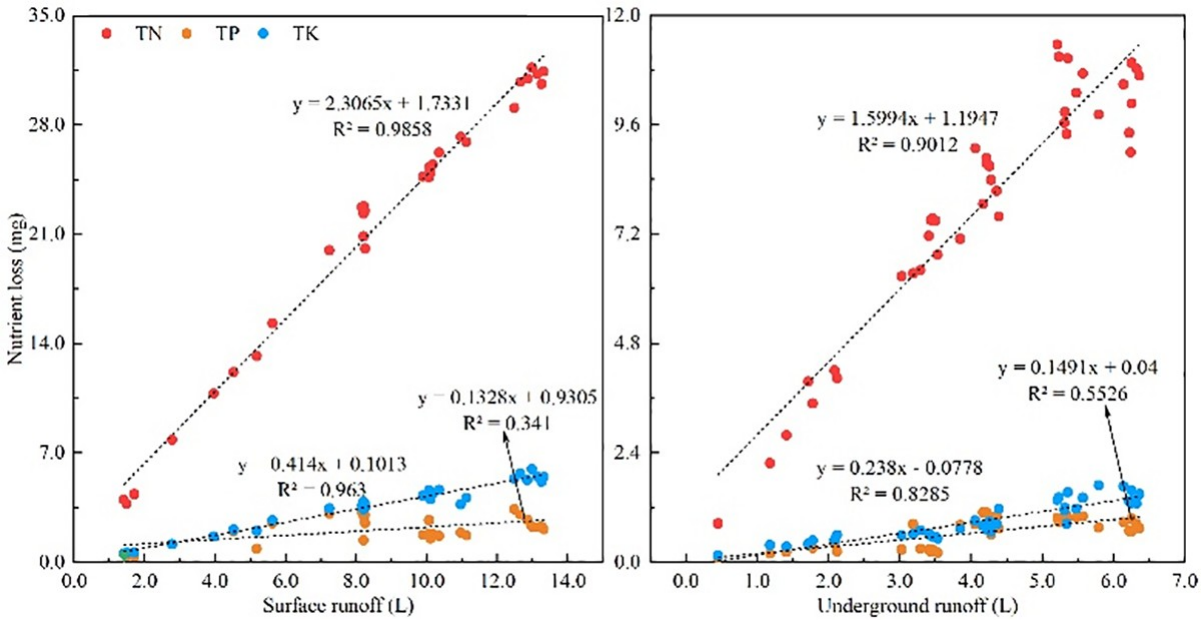
Fitting between nutrient loss and runoff.

Rainfall is the primary source of runoff on sloping farmland. Soil nutrients can be dissolved in the water, and runoff can drain to the surface or underground. The correlation between the loss of nutrients through runoff and rainfall intensity was analyzed to further explore the relationship between rainfall intensity and runoff and nutrient loss. It is evident from Table 4 that the surface runoff, TN loss, TK loss, and rainfall intensity were significantly positively correlated, with correlation coefficients of 0.987, 0.914, and 0.923, respectively; surface runoff was also significantly positively associated with TN loss and TK loss. There was no significant relationship between TP loss and rainfall intensity or between TP loss and surface runoff. Rainfall intensity and underground runoff were significantly positively correlated, but there was no meaningful relationship between underground runoff and nutrient losses. Underground runoff and covert nutrient losses showed a significant positive correlation (Table 5). The nutrient (TN, TP, TK) losses from runoff and runoff were linearly fitted (Fig 9), and TN, TK, and runoff were significantly positively correlated (*P*<0.05). The surface R^2^ values for TN and TK were 0.9856 and 0.9630, respectively. The underground R^2^ values for TN and TK were 0.9012 and 0.8285, respectively, but the surface fitting was better, and the fitting effect of TP was low (the surface and underground R^2^ values were 0.3410 and 0.5526, respectively).

**Table 5:** Correlation analysis of nutrient loss, rainfall intensity, and runoff.

position	index	Surface				underground			
		Runoff	TN	TP	TK	Runoff	TN	TP	TK
Surface	Rainfall intensity	0.978**	0.914**	0.684	0.923**				
	Runoff	1	0.993**	0.584	0.981**				
	TN		1	0.640*	0.984**				
	TP			1	0.659				
	TK				1				
Underground	Rainfall intensity					0.687*	0.559	0.590	0.774
	Runoff					1	0.949**	0.743*	0.910*
	TN						1	0.770*	0.863**
	TP							1	0.692*
	TK								1

**. Significant correlation at 0.01 level (bilateral)

*. Significant correlation at 0.05 level (bilateral).

## Discussion

### Influence of rainfall intensity on runoff in karst sloping farmland

Rainfall intensity is an essential factor affecting runoff, and its magnitude will directly affect the amount of runoff [34]. Nutrients can dissolve in water and enter surrounding reservoirs or groundwater systems as water flows, causing water pollution and harming human production and life. Therefore, studying the characteristics of water flow on sloping farmland with mild rocky desertification in karst areas reveals the law of soil nutrient loss on sloping farmland and provides theoretical support for preventing and controlling the risks caused by nutrient enrichment. Due to the geological structure of karst areas, carbonate rocks on sloping farmland will form karst fissures, sinkholes, and other channels under long-term hydraulic erosion, gravity erosion, chemical erosion, and multiple other forms of decay [35]. As a result, when rainwater contacts sloping farmland, it forms surface runoff down the slope and underground runoff down through such channels. The study results show that under light rainfall intensity (30 mm·h^-1^) on karst sloping farmland, there is no runoff on the sloping surface, and water flows from the soil gap down through the cracks underground, which is in line with [30]. This lack of surface runoff may occur because the soil infiltration capacity is greater than the runoff under light rainfall intensity. The rainfall will enter the soil through infiltration and move downward after the soil is saturated, resulting in no surface runoff. When the rainfall intensity increases to 50 mm·h^-1^, both the surface and underground will generate runoff. As the rainfall intensity increases, the soil surface water content gradually increases, and the soil infiltration capacity decreases. When the soil infiltration rate is equal to or lower than the runoff generated by the rainfall intensity, the surface begins to produce runoff, indicating that the critical rainfall intensity to change from underground runoff to surface runoff in karst sloping farmland may be between 30 and 50 mm·h^-1^.

With the increase in rainfall intensity, both surface and underground runoff increased significantly, but the loss of surface runoff was considerably more significant than that of underground runoff. This difference in runoff loss may be because the kinetic energy of raindrops contacting the soil surface increases with increasing rainfall intensity. Small rills are likely to be formed on the slope as the impact force increases. Most of the rainwater is unable to infiltrate. The formation of super permeable runoff will significantly increase surface runoff. On the other hand, raindrop splashing destroys the structures of surface soil particles, making them challenging to form. The lost crust layer and surface soil crust can reduce water penetration into the ground to a certain extent, resulting in higher surface runoff than underground runoff [36]. This study also found that at approximately 12 minutes of rainfall, the runoff under various rainfall intensities reached its peak. Therefore, in seasons with frequent rain, increasing vegetation coverage and crop residues could be adopted to reduce the direct impact of rainfall on the soil surface. Second, ditches could be dug to make runoff flow away along specific channels to mitigate the effects of rainwater and soil time to control nutrient loss.

### Effect of rainfall intensity on runoff nutrients in karst sloping farmland

Rainfall is the power source of nonpoint source pollution, and the runoff formed is the carrier and solvent of nitrogen and phosphorus pollutant output [26]. The loss of soil nutrients from karst sloping farmland reduces the decline in land productivity. On the other hand, nutrient losses underground through channels such as pores and fissures result in groundwater pollution. Tang et al. [37] determined that the peak concentration of nitrogen and phosphorus loss occurred with the initial scouring effect. The study results revealed that the surface and underground nutrient concentrations under different rainfall intensities showed a gradual downward trend with the extension of rainfall duration but did not show apparent initial erosion effects. The reason for the lack of initial erosion effects may be that the early rainfall time was short; thus, the runoff was not high. Some soluble elements did not dissolve in the rainwater, resulting in little change in the concentration of various nutrients at the beginning of rainfall.

Increases in rainfall intensity reduce the average TN and TK concentrations because runoff can promote the effective dissolution and release of nitrogen and potassium [38]. In the same rainfall period, runoff increases with increases in rainfall intensity. The dilution effect of nitrogen and potassium increased with the addition of runoff. Due to its low solubility, TP is easily absorbed by soil [39]; thus, TP has a weak migration capability in runoff. Therefore, the concentration of TP does not change significantly with rainfall. When the concentration of elements in a water body exceeds an absolute value, long-term accumulation will cause eutrophication of the water body. The average concentration of nitrogen in surface runoff under different rainfall intensities reached 2.56 mg/L, which far exceeded the water eutrophication threshold (TN 0.2 mg/L, TP 0.02 mg/L) [40]. Therefore, during agricultural production, the amount of fertilization and the fertilization period should be controlled to reasonable levels to prevent eutrophication of the water body due to long-term nutrient losses from the sloping farmland.

The amount of nutrient loss generally increased initially and then gradually stabilized as rainfall continued (Fig 5). The greater the rainfall amount was, the greater the nutrient loss was, and the difference between the surface and underground nutrient loss was marked. This difference may be related to the form of nutrient loss. Both granular and soluble nutrient forms can be lost through surface runoff, while the nutrients lost underground are mainly soluble. For example, Chen et al. [41] found that nitrogen loss in surface runoff is mostly granular, while the soil flow is mainly dissolved. The amount of nutrients that quickly dissolve in water depends on soil moisture because the moisture content determines the ratio of element dissolution and adsorption. The amount of nutrients released into the runoff in dissolved form increases with increased soil moisture content [38]. Nitrogen and potassium easily dissolve in water, and the greatest amounts are lost in the dissolved state. Surface runoff is significantly greater than underground runoff. Surface runoff accounts for an average of 64.22% of the total runoff (Table 2). This study found that surface runoff was the primary channel for phosphorus loss in karst sloping farmland during the rainy season. The primary forms of failure were granular phosphorus and dissolved phosphorus [42]. Research has shown that approximately 50% of phosphorus is still fixed in farmland one month after fertilization. When runoff washes the topsoil layer, the phosphorus adsorbed and set on the particle surface will also be lost when the topsoil migrates with the runoff. Soil phosphorus transferred by runoff is mainly particulate; it accounts for 70% ~ 80% of the TP loss [43]. In this study, the surface runoff was significantly higher than the underground runoff, and the higher the entrapment capacity of fine sediment particles was, the greater the loss of surface phosphorus.

A large number of studies have shown [44, 45] that runoff and nutrient loss are correlated. Liu et al. [46] further confirmed the relationship between runoff and nitrogen loss, and that study revealed a very significant positive correlation. This study found that runoff and TN and TK losses were significantly positively correlated (Table 5), showing a power function relationship (Fig 7), which is the same as the results of Idowu [47]. The rainfall intensity exhibited a very significant association with surface runoff, TN loss, and TK loss. Although underground runoff exhibited a substantial relationship with various nutrients, the correlation was low. With the increase in rainfall intensity, there were more dissolved nutrients in surface runoff, an increase in nutrient loss, and the formation of crusts on the surface of the middle and late periods of rainfall, which increased the loss of surface nutrients. A decrease in nutrient loss can be achieved by planting high-coverage crops or increasing crop planting density to reduce the surface nutrient loss on sloping farmland [48]. The loss of underground nutrients in sloping karst farmland is more complicated than that of surface nutrients, so the fitting effect is low.

In terms of the loss ratio, the proportion of surface runoff loss was more significant than that of underground runoff loss, and the balance of surface runoff increased with increasing rainfall intensity; however, the loss of nutrients and diversion from underground runoff was approximately 30% of the total loss (Fig 6). Therefore, we should pay attention to the surface nutrient loss and nutrient loss path in underground runoff in the future. Nutrients are stored in underground runoff, and surface vegetation is difficult to use to decrease nutrient loss. On the other hand, nutrients can directly enter the groundwater system through the underground fissure pipeline, causing soil and water pollution. However, this experiment simulated the double-layer surface and underground spatial structure of karst sloping farmland. However, due to artificial simulation limitations, there was a definite gap between the soil medium’s treatment, the floor rock structure, pore and fissure distribution, and bedrock exposure rate. Simultaneously, the slope and bedrock exposure rates involved in the test design were quite simple. In the future, we should conduct fixed-point, qualitative field experimental research, conduct a detailed investigation of the morphology and connectivity of underground fissures, obtain more detailed data, and combine the results with indoor simulation experiments to determine the nutrient loss patterns in karst sloping farmland.

## Conclusion

This study found that surface runoff is the main route of nutrient loss in rainy seasons on karst sloping farmland, and the critical rainfall intensity underground transport from the ground to the surface may be between 30 and 50 mm·h-1. As the rainfall intensity increases, the surface and underground runoff increase, and the runoff difference becomes more substantial with increases in rainfall. Soil nutrient loss on sloping farmland through pores, cracks, and other channels is more likely to occur than ground surface loss. The nutrient concentrations of surface runoff fluctuate in the process of runoff. The nutrient concentrations of TN and TK in surface runoff are significantly higher than those in underground runoff. The nutrient concentrations of underground runoff gradually decrease with increasing runoff duration. The amount of nutrient loss increases with the increase in rainfall intensity, and the rate of soil nutrient loss through the surface is higher than that underground. Rainfall intensity has a significant effect on runoff and TN and TK nutrient loss, while TP loss exhibits little response to rainfall intensity and surface runoff. In the process of controlling soil and water loss and nutrient loss from gently sloping farmland in karst areas, the reasonable control of runoff loss paths is the key to preventing and controlling nutrient loss. By controlling the generation of underground runoff, increasing surface vegetation coverage, and reducing the contact time between rainwater and the soil, nutrient loss control can be achieved.

## Supporting information

S1 File (XLSX)
